# Anxiety symptoms and puberty interactively predict lower cingulum microstructure in preadolescent Latina girls

**DOI:** 10.1038/s41598-022-24803-4

**Published:** 2022-12-01

**Authors:** Dana E. Glenn, Jenna L. Merenstein, Ilana J. Bennett, Kalina J. Michalska

**Affiliations:** 1 Department of Psychology, University of California, Riverside, 900 University Avenue, Riverside, CA 92521 USA; 2 Brain Imaging and Analysis Center, Duke University, Durham, NC USA

**Keywords:** Psychology, Anxiety

## Abstract

Preadolescence is a period of increased vulnerability for anxiety, especially among Latina girls. Reduced microstructure (fractional anisotropy; FA) of white matter tracts between limbic and prefrontal regions may underlie regulatory impairments in anxiety. However, developmental research on the association between anxiety and white matter microstructure is mixed, possibly due to interactive influences with puberty. In a sample of 39 Latina girls (8–13 years), we tested whether pubertal stage moderated the association between parent- and child-reported anxiety symptoms and FA in the cingulum and uncinate fasciculus. Parent- but not child-reported anxiety symptoms predicted lower cingulum FA, and this effect was moderated by pubertal stage, such that this association was only significant for prepubertal girls. Neither anxiety nor pubertal stage predicted uncinate fasciculus FA. These findings suggest that anxiety is associated with disruptions in girls’ cingulum white matter microstructure and that this relationship undergoes maturational changes during puberty.

## Introduction

Anxiety disorders are a prevalent mental health concern in the United States^1^, with an estimated one-third of girls experiencing an anxiety disorder by adolescence^2^. Greater vulnerability for anxiety symptoms in girls begins in middle childhood (6–12 years) and increases into adolescence (13–18 years)^3–5^ when youth undergo major puberty-related structural brain changes, including in white matter tracts critical for emotion processing and regulation^6–12^. However, the limited developmental work examining white matter microstructure in anxiety is mixed^13–15^ and no studies to our knowledge have focused on minoritized youth. Thus, the current study sought to examine associations among anxiety symptoms, pubertal status, and white matter microstructure in a community sample of Latina girls—a demographic group with elevated anxiety symptoms^16–20^.

A characteristic feature of anxiety disorders is a reduced ability to downregulate negative emotions^21–23^, which is thought to be partially mediated by white matter tracts that facilitate communication between limbic (e.g., amygdala) and emotion regulatory regions in the lateral and medial prefrontal cortex (PFC). Two such white matter tracts are the cingulum, a large-scale fronto-limbic tract that runs in the cingulate gyrus and connects the cingulate cortex with medial prefrontal, parietal, and temporal regions; and the uncinate fasciculus, a smaller-scale fronto-limbic tract that projects from anterior temporal to prefrontal and orbitofrontal regions^24–28^. Diffusion-weighted imaging (DWI) studies have reported that adults with anxiety display reduced fractional anisotropy (FA) in these two tracts^24–28^. FA is a diffusion tensor imaging metric that reflects the degree to which water diffusion is restricted, or anisotropic, and indirectly indexes white matter pathway microstructural integrity^29^. Associations between anxiety and white matter microstructure are less systematic in the developmental literature, with researchers observing that anxiety is associated with reductions in uncinate fasciculus FA among adolescents^15^ and preadolescent boys^14^, but not girls^14,30^. In other work, girls’ anxious and depressive (internalizing) symptoms were correlated with reduced cingulum and uncinate fasciculus FA^31^, and the inverse between internalizing symptoms and cingulum FA was stronger for girls than boys^32^. Thus, although these findings support a structural contribution from cingulum and uncinate fasciculus to the well-documented difficulties with emotion regulation in anxiety^33–35^, the association between anxiety symptoms and microstructure in these tracts remains underspecified in preadolescent girls.

Puberty-driven influences on white matter microstructure in early adolescence may alter connections between emotion processing and regulatory regions^12,36^, and influence anxiety symptoms. White matter microstructure of the cingulum and uncinate fasciculus exhibit protracted development throughout adolescence and into adulthood^10,11^ and shows sex differences during puberty^6,12,37^, suggesting that previous work observing anxiety-related sexual dimorphism in FA may be attributable to puberty-specific structural changes. Puberty also spurs changes in the valence of functional connectivity between the amygdala and anterior cingulate cortex (ACC), regions that are connected by the cingulum, with children exhibiting positive connectivity and adolescents and adults exhibiting negative connectivity^38,39^. This association is moderated by anxiety, such that age is negatively related to amygdala-ACC functional connectivity in healthy youth^38,40,41^ but positively related in anxious youth^40^. As preliminary work in preadolescent girls suggests that there are longitudinal changes in the association between anxiety and cingulum FA^13^ (prior to multiple comparison correction), it is also possible that puberty and anxiety interact to predict cingulum microstructure. However, few studies to date have tested whether pubertal stage moderates the association between anxiety and white matter development.

In a sample of Latina girls aged 8–13 years, we first examined associations between anxiety symptoms (parent- and child-reported) and FA in the cingulum and uncinate fasciculus. We then tested whether anxiety-FA relationships were moderated by pubertal stage. Based on prior findings in the cingulum^13,14,40,42,43^, we hypothesized that anxiety symptoms will be associated with decrements in cingulum microstructure. We further hypothesized that pubertal stage will influence the association between anxiety and cingulum FA, but given the nascent evidence base on puberty effects on cingulum FA^36^, we did not make specific directional predictions. To extend prior work on uncinate fasciculus microstructure in anxious preadolescent girls^13,14,31^, we also tested independent and interactive influences of anxiety and pubertal stage on uncinate fasciculus FA. Because of prior mixed findings, we considered these analyses exploratory.

## Methods

### Participants and procedures

Fifty-five Latina girls were recruited from the Inland Empire region of Southern California to participate in the first wave of a longitudinal study on emotional development. Girls aged 8–13 years were initially eligible to participate if they were medication-free, reported no contraindications for neuroimaging (e.g., no ferrous metal in the body, not pregnant, not claustrophobic), were not experiencing active medical problems or suicidal ideation, and were free from a current psychiatric diagnosis of Tourette’s syndrome or obsessive–compulsive disorder and lifetime history of mania, psychosis, or pervasive developmental disorder. Although menstruation was initially used as an exclusionary criterion, it was later dropped to increase sample size and two postmenarchal participants were recruited.

Across two visits, participants completed a laboratory testing session and magnetic resonance imaging (MRI) data collection. During the laboratory session, children and their caregivers completed a battery of self-report questionnaires assessing demographics, behavior, anxiety, and other mental health outcome measures not reported here. During the MRI session, children underwent T1-weighted and DWI data acquisition. DWI scans were not collected from 16 participants because they did not return for the second visit (*n* = 12), or due to participants’ distress (*n* = 2), time constraints (*n* = 1), or inability to continue data collection during the COVID-19 pandemic (*n* = 1), resulting in a final sample of 39 participants.

Because of our larger focus on social determinants of mental health in Latina youth, children also had to have at least 50% Latinx heritage and self-identify as Latina or Hispanic in order to participate. Our sample consisted of 26 Mexican–American girls, 8 girls of other, mixed, or unspecified Latinx descent, and 5 girls of mixed racial descent (white and Latina). See Table 1 for demographic information.

**Table 1:** Sample demographic characteristics and descriptive statistics for study variables.

N	Age	% female	Pubertal stage (tanner scale)	Household income	Anxiety symptoms (SCARED)	
					Parent-report	Child-report
39	10.1 (1.2)	100	1.9 (0.8)	$64,474 ($45,778)	18.6 (13.7)	37.6 (15.5)

Variables are displayed as mean (standard deviation). *SCARED* Screen for Child Anxiety Related Disorders.

At the end of each visit, participants were compensated with a gift card and a small prize. Study procedures were approved by the University of California, Riverside Institutional Review Board and written informed consent and assent were obtained at the start of the first visit from parents and children, respectively.

### Measures

#### Pubertal staging

Girls’ pubertal stage was measured via self- and parent-assessed Tanner staging^44^. Meta-analytic evidence has found that Tanner staging is a more sensitive predictor of internalizing psychopathology for girls than hormone levels, subjective reports of pubertal timing, or age at menarche^45^. The female version of the Tanner stage line drawings consists of five drawings depicting breasts and five drawings depicting pubic hair. For each set, girls and their caregivers selected the image that reflected the child’s current stage of development. Scores range from 1 (prepubertal) to 5 (postpubertal). An overall pubertal stage composite was created by averaging child- and parent-reported breast and pubic hair development. Scores for dyads with a missing informant report (*n* = 3 children, 1 parent) were composed of the remaining informant’s scores.

#### Anxiety symptoms

**Figure 1:**
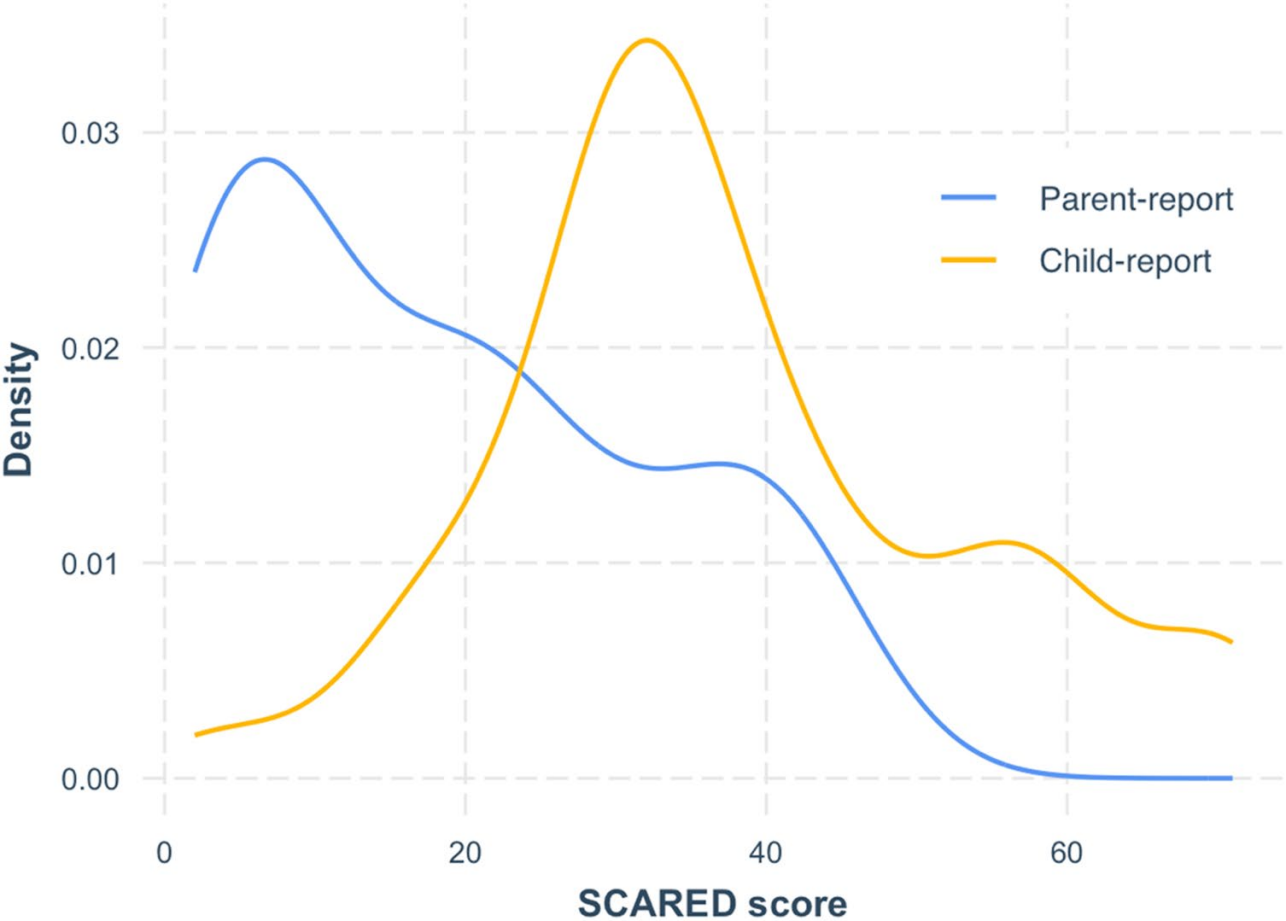
Density distribution of parent- and child-reported scores on the Screen for Child Anxiety Related Disorders (SCARED). Shapiro–Wilk statistical testing for normality revealed skewed distribution of the parent-report (*p* = 0.002) but not child-report (*p* = 0.11). Parent-reported SCARED scores were log-transformed to reduce data skewness.

Children’s anxiety symptoms were collected via self- and parent-report on the Screen for Child Anxiety Related Disorders (SCARED). The SCARED included 41-items assessing recent anxiety symptoms (past 3 months) rated on a 3-point Likert scale and it has strong psychometric properties^46,47^. Item scores were summed to a total score (range: 0–82). Missing parent-report was imputed with mean-replacement (*n* = 1). The normality of parent- and child-reported SCARED were tested with the Shapiro–Wilk test^48^ and by visual inspection of skewness (Fig. 1). Parent-reported SCARED scores were log-transformed to reduce a positive skew in raw scores (*p* = 0.002). Child-reported SCARED scores were normally distributed (*p* = 0.11) and were thus not transformed. The threshold for clinically-significant levels of anxiety (≥ 25) was met in 33 child-reported scores and 13 parent-reported scores.

Children and caregivers may provide distinct information about children’s anxiety symptoms. Child-report on the SCARED has predicted longitudinal changes in whole-brain white matter microstructure^13^, which may be because children are more knowledgeable about feelings of anxiety not externally observable to their parents. Conversely, parent-report on the SCARED has demonstrated higher sensitivity, higher specificity, and matches more closely with clinical cutoffs than child-report^49,50^. Thus, we implemented two separate models with parent-and child-reported anxiety symptoms. We conducted Bonferroni corrections for multiple comparisons across two informant groups (child-report, parent-report) and two regions of interest (cingulum, uncinate fasciculus), (*p* < 0.0125).

#### Imaging data

##### Acquisition

Whole-brain neuroimaging data were collected using a 3 T Siemens Prisma scanner and 32-channel receive-only head coil. A high-resolution T1-weighted scan of the whole-brain was acquired using a magnetization prepared gradient echo sequence (MP-RAGE) with the following parameters: TE/TR = 2.72/2400 ms, flip angle = 8, FOV = 256 × 240 mm, matrix = 320 × 300, voxel size = 0.8 mm^3^, and scan time = 6:28.

A single diffusion-weighted single-shot spin-echo, echo planar imaging (EPI) image was acquired with the following parameters: TE/TR = 102/3500 ms, FOV = 218 × 187 mm, matrix size = 128 × 110, voxel size = 1.7 mm^3^, multiband factor = 4, 72 slices with no gap, and scan time = 8:42. Bipolar diffusion-weighting gradients were applied in 64 directions with b values of 1500 s/mm^2^ and 3000 s/mm^2^ with six b = 0 images.

##### Processing

For each participant, non-brain tissue was removed and a whole-brain mask was generated using Analysis of Functional NeuroImages (AFNI)^51^. Next, we corrected for head movement, EPI distortions, and eddy-current induced distortions using the TOPUP and EDDY commands in FMRIB's Software Library (FSL; www.fmrib.ox.ac.uk/fsl). Finally, FSL’s DTIFIT was used to estimate a single diffusion tensor for each voxel, using data from both b values, with the whole-brain mask limiting tensor fitting to brain tissue. The output included a voxel-wise image for FA.

##### Regions of interest (ROI)

ROI creation followed a pipeline that has been successfully implemented by our group^52–54^. Standard bilateral masks of the cingulum and uncinate fasciculus were first created from the JHU ICBM-DTI-81 white matter labels atlas in FSL^55^. For each participant, the standard cingulum and uncinate fasciculus white matter masks were aligned to native diffusion space using the following registration steps: (1) the MP-RAGE image was aligned to the Montreal Neurological Institute (MNI) 152 1 mm^3^ resolution standard image using an affine transformation with 12 degrees of freedom, (2) the preprocessed diffusion image with no diffusion weighting applied (i.e., my_hifi_b0) was aligned to the MP-RAGE image using a boundary-based registration with six degrees of freedom, (3) the diffusion-to-MP-RAGE and MP-RAGE-to-MNI transformations were concatenated, (4) the concatenated transformation was inverted, and (5) the inverted transformation was applied to align the standard JHU ICBM white matter masks to native diffusion space. The first author (D.G.) visually inspected cingulum and uncinate fasciculus alignment and mask coverage and confirmed that all masks were of usable quality.

Prior to extracting FA values, gray matter tissue and cerebrospinal fluid were excluded from the native space masks by multiplying them by a white matter mask generated for each participant from their MP-RAGE image via FSL’s Automated Segmentation Tool (FAST)^56^. The partial volume estimate of this white matter mask was thresholded at 0.5, aligned to diffusion space by applying the inversion of the diffusion-to-MP-RAGE transformation described above, and multiplied by the bilateral cingulum and uncinate fasciculus masks. The resulting masks were then separately multiplied by each participant’s voxel-wise dtifit_FA image and FA values were averaged across voxels separately within each mask. The volume of each mask, measured as the number of voxels, was also extracted using fslstats.

### Data analysis

To examine the effects of pubertal staging and anxiety symptoms on white matter microstructure, we implemented multiple linear regression analyses in RStudio^57^. Anxiety symptoms and pubertal stage were tested as independent and interactive predictors of ROI FA. To test the relations among anxiety symptoms, pubertal stage, and white matter microstructure independent of individual differences in white matter volume, all analyses controlled for ROI volume. We tested each informant type (child-report, parent-report) and ROI (cingulum, uncinate fasciculus) in four separate models. Pubertal stage and age were strongly correlated in our sample (*r* = 0.65, *p* < 0.001), so we did not include age as a covariate in our model to avoid multicollinearity. Exploratory analyses were conducted to determine whether significant effects of pubertal stage were confounded by age or pubertal timing, the relative measure of pubertal development compared to same-age, same-sex peers^58^ (see Supplementary Materials). To probe significant interactions, simple slopes were examined to assess predictions of white matter microstructure from anxiety symptoms across three levels of pubertal staging (− 1 SD, 0 SD, + 1 SD). No outliers were observed in children’s anxiety symptom scores or FA values (± 3 SD).

### Ethics approval and consent to participate

Study procedures were approved by the University of California, Riverside Institutional Review Board (Study HS-17-208) and all experiments were performed in accordance with relevant guidelines and regulations.

## Results

Linear regression models tested whether anxiety symptoms and pubertal stage interacted to predict FA in each ROI (cingulum, uncinate fasciculus), separately for parent- and child-reported SCARED anxiety scores. Parent-reported SCARED scores were log-transformed to reduce data skewness (see “Methods”).

**Figure 2:**
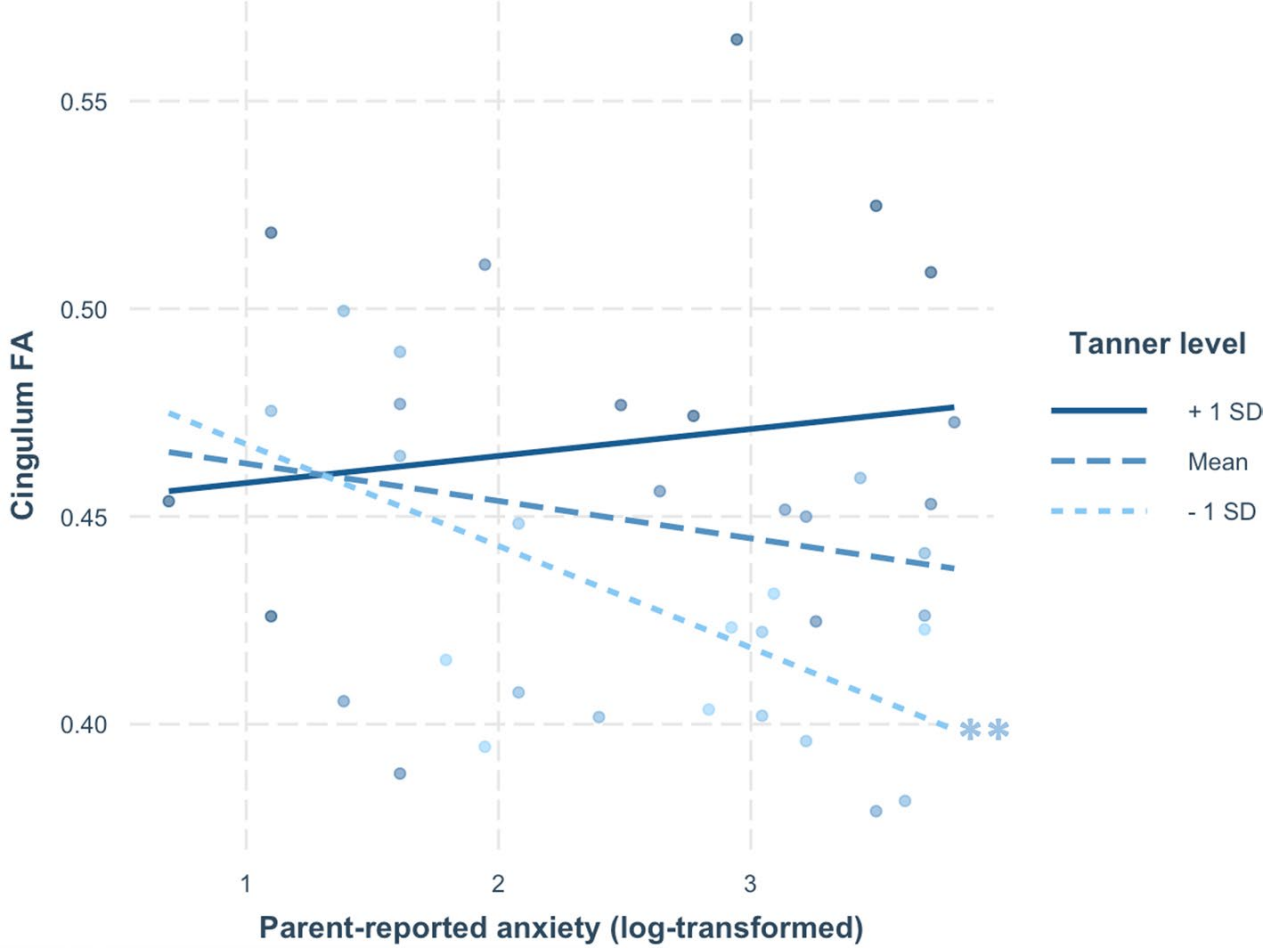
Pubertal stage moderates the association between anxiety symptoms and cingulum FA. Simple slopes tested the association between anxiety symptoms and cingulum FA at Tanner levels of 1.14 (− 1 SD), 1.94 (0 SD), and 2.73 (+ 1 SD). Anxiety symptoms predicted reduced cingulum FA for children in early pubertal stages (− 1 SD). **p < 0.01.

When cingulum FA was the outcome variable (M_FA_ = 0.45 ± 0.04), the model that included log-transformed parent-reported anxiety symptoms and pubertal stage as predictors was significant, *R*^2^ = 0.663, *F*(4, 34) = 16.78, *p* < 0.001. Higher levels of parent-reported anxiety symptoms predicted lower cingulum FA, *p* = 0.001, and this effect was moderated by pubertal stage, *p* = 0.001 (Table 2). Cingulum volume also significantly positively predicted cingulum FA, *p* < 0.001. Follow-up simple slopes analyses revealed that children's parent-reported anxiety symptoms were significantly associated with reduced white matter microstructure in the cingulum at early pubertal stages (Tanner = 1.14), *β* =  − 0.02, *t* =  − 3.24, *p* = 0.003, and not significantly associated with FA at mean (Tanner = 1.94), *β* =  − 0.01, *t* =  − 1.81, *p* = 0.08, and late pubertal stages (Tanner = 2.73), *β* = 0.01, *t* = 1.14, *p* = 0.26 (Fig. 2). Volume was the only predictor of FA in the cingulum when this model was repeated with child-reported anxiety symptoms in place of parent-reported anxiety symptoms (*p* = 0.001), and no other main effects or interactions emerged (*p*s > 0.19). All significant cingulum effects survived Bonferroni correction for multiple comparisons across informants and regions, *p* < 0.0125.

**Table 2:** Statistics are shown for each predictor (*SE*, *t*, *p*) and the overall model (*R*^2^, *p*) demonstrating interactive influences of pubertal stage and log-transformed parent-reported anxiety symptoms on fractional anisotropy (FA) in the cingulum, but not uncinate fasciculus.

		*SE*	*t*	*R*^2^	*p*
**Cingulum FA**					
	Anxiety	0.001	− 3.55		0.001***
	Pubertal stage	0.001	− 1.69		0.10
	Pubertal stage × anxiety	< 0.001	3.47		0.001***
	Cingulum volume	0.005	4.31		< 0.001***
	Overall model			0.664	< 0.001***
**Uncinate fasciculus FA**					
	Anxiety	0.025	− 0.30		0.76
	Pubertal stage	0.028	− 0.11		0.91
	Pubertal stage × anxiety	0.10	0.47		0.64
	Uncinate fasciculus volume	< 0.001	2.02		0.05*
	Overall model			0.172	0.16

For visualization, the standard JHU-ICBM hippocampal cingulum is overlaid on a sagittal view of the standard superior cingulum using a MNI 152 brain (1 mm^3^ resolution) as the underlay. *p ≤ 0.05, **p ≤ 0.01, ***p ≤ 0.001.

When uncinate fasciculus FA was the outcome variable (M_FA_ = 0.43 ± 0.05), no significant effects of anxiety symptoms or pubertal stage emerged for either informant report (*p*s > 0.63). In both models, uncinate fasciculus volume positively predicted FA (*p*s ≤ 0.05) but did not survive Bonferroni correction.

Exploratory analyses testing the influence of age and pubertal timing (residualized pubertal stage) on white matter microstructure in place of pubertal stage did not reveal any significant effects of anxiety symptoms, age, or pubertal timing (see Supplementary Material). This suggests that the significant effects of parent-reported anxiety symptoms and pubertal stage were not due to individual differences in age or pubertal timing.

## Discussion

The present study leveraged diffusion-weighted imaging to examine anxiety symptoms and pubertal stage as interactive predictors of cingulum and uncinate fasciculus white matter microstructure in preadolescent Latina girls. We observed that parent-reported child anxiety symptoms predicted reduced FA in the cingulum but not the uncinate fasciculus. Further, the association between anxiety symptoms and cingulum microstructure was moderated by pubertal stage, such that only prepubertal girls (− 1 SD; Tanner = 1.14) displayed an inverse association between anxiety symptoms and cingulum FA. We suggest that pediatric anxiety symptoms manifest as disruptions in cingulum white matter microstructure in preadolescent girls and identify maturational moderators of this microstructure. Our results were specific to girls and highlight the importance of examining sexes separately when measuring associations between anxiety, pubertal status, and brain development.

Our first key finding was that elevated parent-reported child anxiety symptoms (SCARED) predicted decrements in white matter microstructure in the cingulum but not in the uncinate fasciculus. Anxiety symptoms have been associated with reduced cingulum FA in adults^24,28^ and children^14,30,31,43^. However, developmental studies of cingulum microstructure tested primarily white, high socioeconomic samples of treatment-seeking youth, and several of these findings did not hold for multiple comparison corrections^14,30^. The present study replicated the association between childhood anxiety and reduced cingulum FA and extended these findings to preadolescent Latina girls. Elucidating markers of anxiety in Latinx youth is critical because an emerging evidence base indicates that Latinx youth experience elevated anxiety compared to other ethnic groups^16,17,19,20,59^, which is likely exacerbated by economic or acculturative stress^60–62^ and barriers to treatment access^63–66^. Indeed, 33% of children in our community sample met clinical thresholds for an anxiety disorder (≥ 25 SCARED score) across both informant reports. Identifying reduced cingulum microstructure as a vulnerability marker for pediatric anxiety in this high-risk population may inform and increase the generalizability of diagnosis and treatment. For example, because white matter microstructure exhibits plasticity in youth and can be altered by training^67–70^, targeting cingulum microstructure may be a promising cross-cultural approach for the treatment for pediatric anxiety.

Lower cingulum microstructure is associated with reduced use of emotion regulatory strategies^34^ and emotional dysregulation^35^. The cingulum stretches from the orbitofrontal cortex, along the dorsal surface of the corpus callosum, and down the temporal lobe and is broadly implicated in cognitive control, attention, and emotion regulation^35,71–74^, all of which are disrupted in pediatric anxiety^75–79^. Thus, reduced FA in the cingulum could reflect underlying neural makers of pediatric anxiety, including impaired downregulation of amygdala hyperactivity by regions like the ACC, dorsolateral PFC, and ventromedial PFC^78,80–87^ and reduced amygdala-prefrontal functional coupling^81,88–90^. Convergent evidence suggests a positive association between white matter microstructure in fronto-limbic white matter tracts and cognitive reappraisal, a form of emotion regulation, as well as an inverse association with anxiety traits^34^. Consistent with these prior reports, the current study showed reduced cingulum white matter microstructure in Latina girls with higher anxiety symptoms. Plausible mediators of this association include impaired emotion regulation and cognitive control reflecting diminished recruitment of PFC subregions and ensuing microstructural differences in tracts subserving them. In other words, inefficient recruitment of regulation strategies in anxious preadolescent girls may alter neural communication and affect microstructural properties within important white matter pathways including the cingulum^43^. Future work combining DWI and task-related functional imaging could further elucidate the dynamics between cingulum microstructure and limbic-prefrontal functional connectivity in anxious children.

In the present study, only parent-reported anxiety symptoms predicted cingulum FA, whereas child-reported anxiety was not significantly associated with white matter microstructure in either tract of interest. This contrasts with a study by Aggarwal and colleagues^30^, who observed that child- but not parent-reported SCARED scores were associated with whole-brain and uncorrected cingulum FA in preadolescent girls. However, other studies have observed associations between parent-reported internalizing symptoms and cingulum FA via alternative measures^31,43^. Parent- and child-reported anxiety symptoms often diverge because children tend to report more severe symptoms than parents^91,92^, and this discrepancy may be larger for healthy children^50^. Thus, parents may more accurately estimate clinical cutoffs for anxiety severity compared to children^49,50^. Conversely, children may be more knowledgeable about feelings of anxiety not externally observable to their parents. Our findings underscore the importance of testing both child and parent reports and highlight the need for future work to probe sources of disagreement between informants.

Although potentially underpowered, we also observed that the association between anxiety symptoms and cingulum FA was moderated by pubertal stage, such that an inverse association only emerged for prepubertal girls. Microstructural integrity in the cingulum increases from childhood to adulthood^11^ and is longitudinally associated with girls’ testosterone levels^37^ and pubertal timing^36,93^. Of note, both baseline anxiety symptoms and longitudinal increases in symptoms predict reductions in preadolescent girls' cingulum microstructure^30,42,43^, suggesting that protracted maturation of cingulum microstructure may correspond with changes in pediatric anxiety symptoms. Simple slopes analyses revealed that anxiety symptoms predicted cingulum microstructure for prepubertal girls, but not those who were in early or middle stages of pubertal development. Anxiety may be a particularly potent risk factor for white matter alterations in prepubertal girls because anxiety disorders that onset earlier in childhood are more chronic and persistent^94,95^ and may therefore have more pronounced implications for neurodevelopment. To be conclusive, this possibility would need to be followed up longitudinally, via comparison of cingulum FA in postpubertal girls with a history of anxiety in prepubescence versus no history of prior anxiety. Alternatively, pubertal timing, a relative comparison of pubertal development with same-age, same-sex peers^58^, may underlie differences in cingulum microstructural properties^36^ in girls with anxiety. However, no main or interactive effects of pubertal timing or age emerged in our sample, suggesting that variations in pubertal timing do not underlie the interactive effects of pubertal stage (see Supplementary Material).

The observed maturational differences in cingulum parallel changes in the function of regulatory networks. Several functional imaging studies have observed age-related compensatory changes in the activation of top-down control networks like the ACC and ventromedial PFC, whereby these networks are underactive in anxious children but become enlisted to mitigate anxiety symptoms in anxious adolescents and adults^40,96^. Similarly, during emotion viewing, amygdala-ACC functional connectivity is reduced in anxious children and increased in anxious adults, relative to their non-anxious counterparts, suggesting a change in valence during adolescence^97^. Under this notion, early anxiety-related structural alterations in the cingulum could increase susceptibility to regulatory impairments that are mitigated by increases in white matter microstructure during puberty. The present study illustrates the importance of examining the association between anxiety and brain structure as a function of puberty, rather than solely including puberty as a covariate, as this association may meaningfully change across pubertal development.

Finally, we did not observe an association between anxiety and FA in the uncinate fasciculus. Uncinate fasciculus microstructure has displayed decrements in boys with anxiety^14^, adolescent girls and boys with anxiety^15^, and girls with internalizing symptoms^31^. However, studies in anxious preadolescent girls have not observed significantly reduced uncinate fasciculus FA^13,14^ and have revealed evidence for the lack of an association^13^, suggesting that girls with anxiety do not display uncinate fasciculus impairments that are observed in boys, older youth, and girls with more broad internalizing symptoms. Sexual dimorphism has been observed in the association between anxiety and uncinate fasciculus FA in preadolescents^14^ and young monkeys^98^, but interestingly, these differences were not explained by age, sex hormones, or the heritability of uncinate fasciculus FA. The mechanisms underlying these sex differences in uncinate fasciculus structure are not known. One possibility is that structural differences reflect sex differences in the prevalence and expression of pediatric anxiety disorders^99,100^. However, given that girls display particularly elevated rates of anxiety^3–5^, there are likely alternative sources of impairment. The uncinate fasciculus, which connects ventral regions of the prefrontal cortex with the amygdala via direct projections through the insula^101^, may be one of multiple pathways that can contribute to the development of anxiety when disrupted. Impairment in other prefrontal-limbic pathways, like the cingulum, may instead underlie anxiety symptoms in girls. Lastly, it is possible that there is an association between girls’ uncinate fasciculus FA and anxiety but that we are underpowered to detect differences. However, given that we were powered to detect cingulum effects, and that other studies with larger samples of girls did not detect associations between anxiety and uncinate fasciculus FA^13,14^, it is unlikely that reduced power is the source of the null effect in our sample.

This study had several strengths that should be noted. First, our diffusion data was acquired with 64 directions and a smaller isotropic voxel size (1.7 mm^3^) than most developmental studies of white matter microstructure in anxiety. These parameters allow for more precise registration of standard regions of interest to native diffusion space, especially for smaller tracts like the uncinate fasciculus. Second, our sample consisted of well-characterized Latina girls—a demographic group that is not well-represented in research, even though Latinx children are one of the largest and fastest-growing ethnic groups in the United States^102^ and experience a higher prevalence of anxiety-related disorders relative to non-Latinx white samples^16–20^.

Despite these strengths, several limitations and future research considerations should also be considered. First, sample size was modest. Modest effects and low replicability have motivated escalating calls to establish consortia-sized samples to identify stable biology-behavior associations^103,104^. Recent reports suggest that brain-behavior effects observed in small studies are sometimes inflated and cannot be replicated in larger samples^105^, contributing to problems with reproducibility. Of note, the reproducibility of behavioral associations with DTI measures of white matter microstructure remains to be assessed. We contend that the current results may help inform future large-scale studies by first identifying effects within a sample of Latina girls, who are underrepresented in extant neuroimaging work. Latinx groups often show reduced participation in research due to barriers to access, including lack of transportation, lack of childcare, and interference with work and family responsibilities^106–108^. As large-scale studies in the United States are largely comprised of non-Latinx white participants^109^, small-sample neuroimaging studies have an important place in building an evidence base linking brain and behavior in under-represented groups for which large samples may be less feasible to obtain. The cost and practical challenges of large-scale recruitment and testing also imply that the time and resources for behavioral phenotyping are limited^110^. To overcome the limitations of under-representation and minimal phenotyping, we have adopted a Community-Based Participatory Research Approach to involve community members in all aspects of our research design, implementation, and dissemination^111^. We also collected dimensional phenotypes (SCARED), which exhibit greater reliability than categorical diagnoses^112^, an approach consistent with the National Institutes of Mental health Research Domain Criteria initiative^113^. Still, effect sizes should be interpreted with caution and current findings should be considered while acknowledging the relatively small sample size. Second, this was a cross-sectional study and the girls in our sample were primarily in early and middle stages of pubertal development due to our initial recruitment of only premenstrual children. Thus, we are limited in our ability to make inferences about developmental processes. Further, it is difficult to disentangle the effects of age and pubertal status, given that they are inherently confounded in all youth samples. Puberty and brain development occur alongside, and are influenced by, the accumulation of experiences with age^114–116^. Increasing exposure to certain stimuli or situations with age may underlie maturational changes in brain structure in any developmental study. However, our finding that age and pubertal timing did not moderate the association between anxiety and FA, even though they were correlated with pubertal stage, indicates that the observed interactive effects were largely driven by pubertal stage. Future studies should include later pubertal stages and, if possible, longitudinally track children’s anxiety symptoms and white matter microstructure throughout puberty and into adulthood. In addition, including measures of hormonal concentration may help address the mechanism by which pubertal development influences cingulum microstructure and why sex differences emerge in the association between anxiety and uncinate fasciculus FA. Finally, anxiety in childhood in minoritized youth may be an index of other psychosocial stressors that were not measured in the present analyses. Factors like acculturative stress and low treatment engagement may contribute to the elevated levels of anxiety in our community sample of children. Future research might continue to examine how psychosocial stressors may mediate the relationship between anxiety symptoms and white matter microstructure among minoritized youth^117^.

In summary, the current study examined the influence of anxiety symptoms and pubertal stage on white matter microstructure in a sample of preadolescent Latina girls. Our results suggest that anxiety contributes to disruptions in cingulum white matter microstructure, and that this relationship varies as a function of pubertal stage. Anxiety-related microstructural vulnerability in white matter regions implicated in cognition and emotion regulation may represent a key biomarker to be targeted in future intervention efforts, especially among preadolescent girls who are at elevated anxiety risk.

## Supplementary Information

Supplementary Information.

## Data Availability

The data in the current study are available from the corresponding author upon reasonable request.
