# Supplementary material for: Anxiety symptoms and puberty interactively predict lower cingulum microstructure in preadolescent Latina girls
Source: Sci Rep. 2022 Dec 1;12:20755. doi: 10.1038/s41598-022-24803-4 (PMC9713745; doi:10.1038/s41598-022-24803-4)
Supplement: Supplementary file 1 — Supplementary Information. [file 41598_2022_24803_MOESM1_ESM.docx]

**Supplementary Material**

Exploratory analyses were conducted to ensure that significant effects of puberty were not driven by age or pubertal timing, the relative measure of pubertal stage compared to peers.

**Age Effects**

Anxiety symptoms and age were tested as independent and interactive predictors of ROI FA, controlling for ROI volume. Each informant type (child-report, parent-report) and ROI (cingulum, uncinate fasciculus) was tested in four separate models. A main effect of ROI volume emerged in all four models, *p*s < .024. A main effect of age on cingulum microstructure emerged in the model with child-reported anxiety symptoms, *p* = .033, but did not hold after Bonferroni correction. No other main effects or interactions emerged in any of the models, *p*s > .08, suggesting that the effects of parent-reported anxiety and pubertal stage on cingulum FA were not due to age differences.

**Pubertal Stage Effects**

To examine the influence of pubertal timing on cingulum FA, we regressed age onto pubertal stage and calculated standardized residual pubertal stage scores. Anxiety symptoms and pubertal timing scores were tested as independent and interactive predictors of ROI FA, controlling for ROI volume. Again, each informant type (child-report, parent-report) and ROI (cingulum, uncinate fasciculus) was tested in four separate models. A main effect of ROI volume emerged in all four models, *p*s < .022. No other significant effects emerged in any of the four models, *p*s > 0.14. This suggests that individual differences in pubertal timing do not underlie variations in cingulum or uncinate fasciculus microstructure in our sample.
